# Changes in Glycemic Parameters Following Total Knee Arthroplasty in Patients With Type 2 Diabetes Mellitus: A Retrospective Observational Study in a South Indian Population

**DOI:** 10.7759/cureus.112826

**Published:** 2026-07-16

**Authors:** Vishnu Senthil, Venkata Vinay Atluri, Karthik Shanmugam, Nithesh J Bensam

**Affiliations:** 1 Orthopaedics and Trauma, Kilpauk Medical College, Chennai, IND; 2 Orthopaedic Surgery, Sri Sri Holistic Hospitals, Hyderabad, IND; 3 Orthopaedics and Rehabilitation, Sri Venkateswara Medical College Hospital and Research Institute, Chennai, IND; 4 Orthopaedics and Trauma, Chettinad Hospital and Research Institute, Chennai, IND

**Keywords:** glycemic control, osteoarthritis, physical activity, total knee arthroplasty, type ii diabetes mellitus

## Abstract

Background

Knee osteoarthritis (OA) is a major cause of disability among older adults and is frequently associated with type 2 diabetes mellitus (DM). Pain and functional limitation in advanced OA may contribute to reduced mobility and poorer glycemic control. Although total knee arthroplasty (TKA) reliably improves pain and function, changes in glycemic parameters following TKA have not been well characterized. This study evaluated changes in glycemic parameters following TKA in patients with type 2 DM.

Methods

A retrospective observational study was conducted in patients who underwent primary TKA for tricompartmental OA between January and November 2016. Patients aged >50 years with established type 2 DM receiving an unchanged antidiabetic regimen throughout follow-up were included. Fasting blood glucose (FBS), postprandial blood glucose (PPBS), glycated hemoglobin (HbA1c), and Lower Extremity Activity Scale (LEAS) scores were assessed preoperatively and at 12 months postoperatively. Paired t-tests were used to compare the preoperative and postoperative outcomes, and Pearson correlation analysis was performed to examine the associations between postoperative LEAS scores and glycemic parameters.

Results

A total of 110 patients met the initial eligibility criteria. Ten patients were excluded from the final analysis because of discontinuation or modification of antidiabetic therapy during follow-up, leaving 100 patients for analysis (46 women and 54 men; mean age, 62 years (range, 55-93)). LEAS scores improved significantly from 7.4±0.9 to 12.8±1.5 (p<0.001). Significant reductions were observed in FBS (139.6±36.1 to 105.5±16.9 mg/dL), PPBS (203.5±40.9 to 149.6±28.8 mg/dL), and HbA1c (7.3±0.9 to 6.6±0.7) (all p<0.001). Postoperative LEAS scores demonstrated significant negative correlations with FBS and PPBS but were not significantly correlated with HbA1c.

Conclusions

In this selected cohort of patients with type 2 DM who remained on an unchanged antidiabetic regimen, glycemic parameters were observed to be reduced and lower extremity functional status improved at 12 months following TKA compared with baseline. These findings represent an observational association rather than evidence of a causal effect and should be interpreted in the context of the retrospective, uncontrolled study design. Prospective controlled studies are needed to determine the independent relationship between TKA and long-term glycemic outcomes.

## Introduction

Osteoarthritis (OA) is a chronic degenerative joint disease and one of the leading causes of disability worldwide [1]. Knee OA in particular contributes significantly to pain, reduced mobility, and impaired quality of life among elderly individuals [2]. Traditionally regarded as a purely degenerative condition, OA is increasingly recognized as a disease influenced by metabolic and inflammatory factors [3].

DM is among the most prevalent non-communicable diseases affecting the elderly population, particularly in developing countries such as India [4]. Increasing evidence suggests a strong association between OA and metabolic disorders, including obesity, insulin resistance, and diabetes mellitus. Adipose tissue functions as an active endocrine organ, producing pro-inflammatory mediators such as tumor necrosis factor-α, interleukin-6, and leptin, which contribute to systemic inflammation and metabolic dysregulation [5].

Patients with advanced knee OA frequently experience pain, deformity, and gait disturbance that significantly limit physical activity. Reduced mobility often leads to sedentary behavior and worsening metabolic health, including poor glycemic control in diabetic patients [6].

TKA is a well-established surgical intervention for advanced knee OA and has consistently been shown to provide substantial pain relief and functional improvement. Restoration of joint alignment and mobility following TKA may enable patients to increase their physical activity levels, thereby positively influencing metabolic parameters [7].

Previous studies evaluating the effect of TKA on glycemic control have reported heterogeneous findings. Al Turki et al. found a trend toward improved HbA1c levels following TKA in patients with type 2 DM, hypothesizing that improved mobility after surgery may contribute to better glycemic control. However, the observed changes were modest and not uniformly sustained across all patients [8]. Conversely, other studies have suggested minimal or no sustained metabolic benefit, highlighting the multifactorial nature of glycemic regulation [9]. Systematic reviews have primarily focused on perioperative complications in diabetic patients rather than long-term metabolic outcomes. Therefore, the relationship between TKA and postoperative glycemic control remains insufficiently defined.

The primary objective of the present study was to evaluate the association between TKA and changes in fasting blood glucose (FBS), postprandial blood glucose (PPBS), and glycosylated hemoglobin (HbA1c) levels in patients with type II DM. As a secondary exploratory analysis, we examined the relationship between postoperative functional status, assessed using the Lower Extremity Activity Scale (LEAS), and postoperative glycemic parameters. LEAS was used as a measure of functional capacity rather than a direct measure of physical activity volume or energy expenditure. We hypothesized that improved postoperative functional recovery may be associated with better glycemic control, while recognizing that this study was not designed to establish a causal or mediating relationship.

## Materials and methods

Study design

This retrospective observational study evaluated patients with primary tricompartmental knee OA and pre-existing type 2 DM who underwent primary TKA at Government Royapettah Hospital, Kilpauk Medical College, Chennai, India, between January and November 2016. The study was approved by the Institutional Ethics Committee (IEC No. 24-03/2017). Owing to the retrospective study design, the requirement for informed consent for data analysis was waived. However, all patients had provided written informed consent at the time of surgery permitting the use of anonymized clinical data for research purposes.

Patient selection

Hospital records were retrospectively reviewed to identify eligible patients. A total of 110 patients fulfilled the eligibility criteria.

Inclusion criteria

The inclusion criteria were: patients aged >50 years; primary tricompartmental knee OA treated with primary TKA; established type 2 DM receiving medical treatment; and unchanged antidiabetic medication regimen throughout the 12-month follow-up period.

Exclusion criteria

The exclusion criteria were as follows: Patients requiring modification or escalation of antidiabetic medication during follow-up; presence of significant comorbidities affecting metabolic status, including chronic kidney disease (Stage ≥3); chronic liver disease, uncontrolled thyroid disorders, active malignancy, chronic corticosteroid therapy, systemic inflammatory diseases (e.g., rheumatoid arthritis), and incomplete follow-up data.

Patients whose antidiabetic medication regimen changed during follow-up were excluded from the final analysis to minimize pharmacological confounding. This exclusion criterion may have introduced selection bias and is acknowledged as a study limitation.

Surgical technique and postoperative rehabilitation

All patients underwent primary TKA using a standardized institutional surgical protocol performed by experienced arthroplasty surgeons. Perioperative care, including anesthesia, antibiotic prophylaxis, thromboprophylaxis, and postoperative analgesia, followed institutional protocols. Postoperative rehabilitation was standardized for all patients and included early mobilization beginning on the first postoperative day, supervised physiotherapy emphasizing knee range-of-motion exercises, quadriceps strengthening, gait training, and progressive weight bearing as tolerated. Patients attended scheduled outpatient follow-up visits according to institutional practice.

Data collection

Clinical records were reviewed retrospectively. FBS, PPBS, HbA1c, and LEAS scores were recorded preoperatively and at the routine 12-month postoperative follow-up. LEAS was used as a standardized measure of postoperative functional capacity and ambulatory status. Although LEAS reflects functional activity, it is not a direct measure of physical activity volume or energy expenditure. Additional variables, including body mass index (BMI), weight change, duration and severity of diabetes, detailed antidiabetic medication class and dosage, dietary modifications, rehabilitation adherence, socioeconomic status, and comorbidity burden, were not consistently available in the retrospective dataset and therefore could not be included in the analysis.

Statistical analysis

All analyses were performed using IBM SPSS Version 27 (IBM Corp., Armonk, NY). Paired t-tests were used to compare preoperative and postoperative values of FBS, PPBS, HbA1c, and LEAS scores. Pearson correlation analysis was performed to assess the relationship between postoperative activity levels and glycemic parameters. A p-value <0.05 was considered statistically significant. Because this was an exploratory retrospective observational study without prespecified primary and secondary hypotheses, statistical significance was defined as a two-sided p<0.05, and no formal adjustment for multiple comparisons was performed. Accordingly, secondary analyses, particularly the correlation analyses, should be interpreted cautiously.

## Results

A total of 110 patients were initially enrolled in the study. Ten patients were excluded at final follow-up due to discontinuation or modification of antidiabetic medication, leaving 100 patients for final analysis. The mean age of the study population was 62 years (range, 55-93 years), with 46 women and 54 men.

At one-year follow-up, significant improvements were observed in both lower extremity functional scores and glycemic parameters. The mean LEAS score increased from 7.4±0.9 preoperatively to 12.8±1.5 postoperatively (paired t-test: t(99)=33.2, p<0.001), indicating a substantial improvement in functional capacity.

FBS decreased from 139.6±36.1 mg/dl to 105.5±16.9 mg/dl (t(99)=9.85, p<0.001), while PPBS decreased from 203.5±40.9 mg/dl to 149.6±28.8 mg/dl (t(99)=11.72, p<0.001). HbA1c improved from 7.3±0.9 to 6.6±0.7 (t(99)=8.91, p<0.001). All changes were statistically significant (Table 1). The mean differences for glycemic parameters were calculated as preoperative minus postoperative values, such that positive values indicate a reduction. For LEAS, the mean difference was calculated as postoperative minus preoperative values, such that positive values indicate improvement in functional activity.

**Table 1:** Comparison of preoperative and one-year postoperative glycemic parameters and physical activity levels in patients undergoing TKA. FBS: Fasting blood sugar; PPBS: postprandial blood sugar; HbA1c: glycosylated hemoglobin; LEAS: Lower Extremity Activity Scale.

	Preoperative (Mean±SD)	At 1 year (Mean±SD)	Mean Diff (Pre-Post)	t	P value
FBS (mg/dl)	139.6±36.1	105.5±16.9	34.1	9.85	P <0.001
PPBS (mg/dl)	203.5±40.9	149.6±28.8	53.9	11.72	P <0.001
HbA1c	7.3±0.9	6.6±0.7	0.7	8.91	P <0.001
LEAS	7.4± 0.9	12.8±1.5	5.4	33.2	P <0.001

Pearson correlation analysis demonstrated a moderate negative association between postoperative physical activity levels and FBS (r=-0.458, t(98)=-5.11, p<0.001), indicating that higher activity levels were associated with lower fasting glucose levels. A weaker but statistically significant negative correlation was also observed with PPBS (r=-0.216, t(98)=-2.20, p=0.030). However, no statistically significant association was found between postoperative physical activity levels and HbA1c (r=-0.143, t(98)=-1.44, p = 0.153) (Table 2).

**Table 2:** Pearson correlation analysis between postoperative LEAS scores and glycemic parameters. Postoperative physical activity demonstrated a significant negative correlation with FBS and PPBS, indicating that higher activity levels were associated with lower glucose levels. However, the correlation with HbA1c did not reach statistical significance (p=0.153). FBS: Fasting blood sugar; PPBS: postprandial blood sugar; HbA1c: glycosylated hemoglobin.

LEAS	FBS	PPBS	HbA1c
r	-0.458	-0.216	-0.143
t	-5.11	-2.20	-1.44
p-value	<0.001	0.030	0.153

## Discussion

DM is one of the most common comorbidities encountered in patients undergoing TKA. With the global rise in both OA and diabetes prevalence, the coexistence of these conditions has become increasingly common in arthroplasty practice.

Several studies have demonstrated that patients with diabetes undergoing TKA are at increased risk of complications, including periprosthetic joint infection, deep vein thrombosis, and hospital readmission [10]. These adverse outcomes are often associated with poor perioperative glycemic control and the systemic inflammatory milieu related to chronic hyperglycemia.

Despite these risks, TKA remains the most effective treatment for end-stage knee OA and consistently provides substantial pain relief and functional improvement [11]. Patients with diabetes frequently present with worse baseline functional status and reduced quality of life due to limited mobility and chronic pain [12].

In the present study, patients demonstrated lower FBS, PPBS, and HbA1c values at the 12-month postoperative assessment compared with baseline, together with significantly improved LEAS scores. These findings represent an observational association and should not be interpreted as evidence of a causal effect of TKA.

Several mechanisms may potentially explain the observed association; however, these mechanisms were not directly evaluated in the present study and therefore remain speculative. Pain relief and restoration of function following TKA may facilitate greater participation in daily activities. Increased physical activity has been associated with improved insulin sensitivity, enhanced skeletal muscle glucose uptake, and reduced systemic inflammation in previous studies [13]. However, because objective measures of physical activity, energy expenditure, and inflammatory biomarkers were not collected, the present study cannot determine whether these mechanisms contributed to the observed glycemic change.

The relationship between OA, metabolic syndrome, and systemic inflammation is increasingly recognized. Adipose tissue acts as an endocrine organ, releasing adipokines and inflammatory mediators such as tumor necrosis factor-α, interleukin-6, and leptin, which contribute to chronic low-grade inflammation and insulin resistance central to type 2 DM [5]. Although reduced adipose burden following improved mobility may represent a plausible pathway for improved glycemic control, this study did not include anthropometric or body composition measurements. Future studies incorporating serial weight and body composition assessments are needed to clarify this relationship.

Correlation analysis in the present study demonstrated a moderate negative association between postoperative functional scores and fasting blood glucose, suggesting that improved functional capacity may contribute to better short-term glycemic control. A weaker association was observed with postprandial glucose, while no significant correlation was found with HbA1c. This indicates that while increased functional capacity may influence immediate glycemic parameters, long-term glycemic control is likely affected by additional factors beyond physical activity alone.

It is important to note that LEAS is a functional grading scale and does not directly quantify physical activity volume or energy expenditure. Furthermore, important postoperative variables not captured in this study, such as adherence to physiotherapy, analgesic or corticosteroid use, pain levels, and patient-reported activity, may independently influence both functional outcomes and glycemic parameters. Future prospective studies should incorporate objective measures of activity, such as accelerometry, alongside validated functional scales.

While most existing literature has focused on the increased perioperative risks in diabetic patients undergoing arthroplasty [8-11], fewer studies have evaluated the potential metabolic benefits associated with improved postoperative mobility. The present findings suggest that improved glycemic parameters may accompany functional recovery following TKA in selected patients with stable diabetes management. Whether this association reflects the effects of surgery itself, postoperative rehabilitation, improved clinical engagement, lifestyle modification, or other unmeasured factors cannot be determined from the present study.

Strengths and limitations

The present study has several strengths. First, it evaluates the potential metabolic benefits of TKA in patients with type II DM, a topic that has been relatively underexplored in the arthroplasty literature. Second, glycemic parameters, including FBS, PPBS, and HbA1c, were objectively measured both preoperatively and at one-year follow-up, allowing for an assessment of both short-term and intermediate-term glycemic control. Third, physical activity levels were evaluated using the validated LEAS, enabling the assessment of the relationship between functional improvement and metabolic outcomes. In addition, to reduce pharmacological confounding, the primary analysis included patients who remained on an unchanged antidiabetic regimen throughout follow-up. However, this exclusion criterion may also have introduced selection bias and is therefore considered a methodological limitation.

However, several important limitations must be acknowledged. First, the one-year follow-up coincides with the period of most active postoperative rehabilitation, and it is possible that improved glycemic parameters partly reflect the heightened clinical attention and structured physical therapy of early recovery rather than a durable metabolic benefit of TKA. An extended follow-up of two to five years would be necessary to determine whether the observed changes are sustained. Second, the retrospective design and absence of a control group preclude causal inference; without a comparator group of diabetic OA patients managed non-operatively, secular trends in diabetes management and regression to the mean cannot be excluded. Third, the study did not differentiate by sex or record menopausal status. Post-menopausal oestrogen deficiency is associated with reduced insulin sensitivity and adipose redistribution, and the failure to account for these factors introduces unmeasured heterogeneity into the cohort. Sex-stratified analyses should be incorporated in future studies. Fourth, BMI and weight data were unavailable, preventing the evaluation of whether adiposity reduction contributed to glycemic improvement. This is a significant omission given the mechanistic framework discussed. Fifth, LEAS is a functional grading instrument, not a direct measure of physical activity volume or energy expenditure; actual postoperative activity levels, physiotherapy adherence, pain scores, and corticosteroid or analgesic use were not captured and represent additional unmeasured confounders. Sixth, the exclusion of patients requiring modification of antidiabetic therapy during follow-up, although intended to reduce confounding, introduces potential selection bias by preferentially including metabolically stable individuals. This may lead to an overestimation of the observed improvement in glycemic parameters and limit the generalizability of the findings to the broader diabetic population undergoing TKA. Seventh, socioeconomic characteristics, dietary modifications, and changes in medication adherence were not available and may have influenced postoperative glycemic outcomes independently of surgery. Eighth, retrospective data abstraction was not independently verified or performed under blinded conditions, introducing the potential for information bias. Ninth, responder-level analyses were not performed; consequently, the study reports only mean changes and cannot determine the proportion of patients whose glycemic control improved, remained unchanged, or worsened following surgery.

Future prospective studies with larger, sex-stratified cohorts, a matched non-operative control group, longer follow-up (two to five years), and objective measurement of physical activity, body composition, and dietary factors are required to determine the independent relationship between functional recovery following TKA and long-term glycemic outcomes after adjustment for important clinical, behavioural, and socioeconomic confounders.

## Conclusions

In this retrospective observational cohort of patients with advanced knee OA and type II DM maintained on stable antidiabetic therapy, glycemic parameters were significantly lower at 12 months postoperatively compared to baseline, alongside improved lower extremity functional scores. These findings represent an observational association that cannot be attributed causally to TKA in the absence of a control group, objective activity measurement, or adjustment for key confounders, including body weight and sex. Future prospective, controlled studies are needed to determine whether restoration of joint function following TKA provides metabolic benefits beyond pain relief and functional improvement.

In this retrospective observational study of patients with advanced knee OA and type 2 DM who remained on an unchanged antidiabetic regimen, FBS, PPBS, and HbA1c values were lower at 12 months following TKA than at the preoperative assessment, accompanied by improved LEAS scores. These findings represent an observational association within a selected cohort and should not be interpreted as evidence of a causal effect of TKA. The observed changes may reflect the combined influence of surgery, structured postoperative rehabilitation, enhanced clinical follow-up, lifestyle modifications, and other unmeasured confounding factors. Prospective controlled studies incorporating objective measures of physical activity, comprehensive assessment of clinical and socioeconomic confounders, and longer-term follow-up are required to determine the independent relationship between TKA, functional recovery, and glycemic outcomes.
